# Peer Review of "Postacute Sequelae of COVID-19 and Adverse Psychiatric Outcomes: Protocol for an Etiology and Risk Systematic Review"

**DOI:** 10.2196/45304

**Published:** 2023-03-14

**Authors:** Dacre Knight

**Affiliations:** 1 Mayo Clinic Jacksonville, FL United States

**Keywords:** COVID-19, long COVID, post–COVID-19 condition, postacute sequelae of COVID-19, PASC, psychiatry, epidemiology, evidence-based medicine

*This is a peer-review report submitted for the paper “Postacute Sequelae of COVID-19 and Adverse Psychiatric Outcomes: Protocol for an Etiology and Risk Systematic Review.”*

## Round 1 Review

### Serious Concerns

*Do you have any serious concerns about the manuscript such as fraud, plagiarism, unethical or unsafe practices?*

No.

*Have authors’ provided the necessary ethics approval (from authors’ institution or an ethics committee)?*

Not applicable.

### Language Quality

*How would you rate the English language quality?*

High quality.

### Validity and Reproducibility

*Is the reasons for conducting the study and its objectives clearly explained?*

Yes.

*Is the study design appropriate?*

Yes.

*Are sufficient details provided so that the method can be replicated?*

Yes.

*Are datasets available so that others could use them?*

Not applicable.

### Suggestions

*Based on your answers in section 3 how could the author improve the protocol?*

There is a more specific definition of PASC that should be included (with a reference). There is a need to list specific medical databases to search and not just mention “various” [1]. PECO criteria need to be listed and not only implied that they will be used.

*Do you have any other suggestions, feedback, or comments for the Author?*

The GRADE approach will be useful, as is mentioned along with a narrative synthesis if needed. Strengths and limitations seem accurate and are good to list.

### Decision

*Verified with reservations:* The content is scientifically sound but has shortcomings that could be improved by further studies and minor revisions.
